# T1. STRESSFUL LIFE EVENTS AND PERCEIVED STRESS IN THE SAMPLE OF PATIENTS WITH FIRST-EPISODE PSYCHOSIS AND HEALTHY CONTROLS: PRELIMINARY RESULTS

**DOI:** 10.1093/schbul/sby016.277

**Published:** 2018-04-01

**Authors:** Anna Butjosa, Regina Vila-Badia, Núria Del Cacho, Itziar Riera-López de Aguileta, Mar Álvarez, Daniel Muñoz, Carme Saltó, Núria Grases, Susana Ochoa, PROFEP Group, Judith Usall

**Affiliations:** 1 Parc Sanitari Sant Joan de Déu, University of Barcelona; 2 Fundació Sant Joan de Déu; 3 Parc Sanitari Sant Joan de Déu; 4 Hospital Sant Joan de Déu

## Abstract

**Background:**

Despite the evidence related to the role of major life events and childhood trauma in the development of first-episode psychosis (FEP; Varese et al., 2012; Morgan & Fisher, 2007), there are few studies on environmental exposure to stressful life events (SLEs) and how SLEs might influence the onset of a psychotic disorder, and the role of perceived stress in this population. The proposed analyses will investigate the association between the categories of SLEs (education, work, partner, family, home, legal, finances, social and health) and perceived stress between patients with FEP and healthy controls (HC).

**Methods:**

Participants were patients with FEP (n=15) and HC (n=21). This research was part of a longitudinal observational study called the ‘PROFEP group’ in Catalonia. Stressful life events were assessed with the Questionnaire of stressful life events (QSLE) (Butjosa et al., 2017). We analysed the frequency of the categories of SLEs. Perceived stress was assessed with the Perceived Stress Scale (PSS; Cohen & Williams, 1988).

**Results:**

There are more frequency of SLEs in the education (p<0.05) and health (p<0.05) categories, and perceived stress (p<0.05) in FEP sample than HC.

**Discussion:**

Results show the relevance of the presence of SLEs (e.g. education and health) and a potent source of perceived stress in FEP sample. Therefore, more studies are needed to evaluate these stressors to apply future psychological interventions in relation to stress management in FEP population. In addition, it would add protective variables in the analyses such as resilience, coping and social support.

